# First evidence for postzygotic reproductive isolation between two populations of Eurasian perch (*Perca fluviatilis *L.) within Lake Constance

**DOI:** 10.1186/1742-9994-5-3

**Published:** 2008-01-24

**Authors:** Jasminca Behrmann-Godel, Gabriele Gerlach

**Affiliations:** 1 Limnological Institute, University of Konstanz, Mainaustraße 252, 78457 Konstanz, Germany; 2 Zoodiversity and Evolution, Carl von Ossietzky University Oldenburg, 26111 Oldenburg, Germany

## Abstract

**Background:**

The evolution of reproductive traits, such as hybrid incompatibility (postzygotic isolation) and species recognition (prezygotic isolation), have shown their key role in speciation. Theoretical modeling has recently predicted that close linkage between genes controlling pre- and postzygotic reproductive isolation could accelerate the conditions for speciation. Postzygotic isolation could develop during the sympatric speciation process contributing to the divergence of populations. Using hybrid fitness as a measure of postzygotic reproductive isolation, we empirically studied population divergence in perch (*Perca fluviatilis *L.) from two genetically divergent populations within a lake.

**Results:**

During spawning time of perch we artificially created parental offspring and F_1 _hybrids of the two populations and studied fertilization rate and hatching success under laboratory conditions. The combined fitness measure (product of fertilization rate and hatching success) of F_1 _hybrids was significantly reduced compared to offspring from within population crosses.

**Conclusion:**

Our results suggest intrinsic genetic incompatibility between the two populations and indicate that population divergence between two populations of perch inhabiting the same lake may indeed be promoted by postzygotic isolation.

## Background

One of the central issues of evolutionary debate is whether or not reproductive isolation, which is a prerequisite for any speciation process, can evolve in the absence of physical barriers to gene flow [1-4]. Many theoretical models predict that speciation occurs under sympatric and parapatric conditions [2,5]. The most common models that try to explain sympatric speciation are either based on habitat-race formation, driven by habitat specific deleterious or beneficial alleles [1,6], or on sexual selection, based on assortative mating [7,8]. Disruptive selection among ecotypes followed by assortative mating could result in reduced gene flow between populations [9-14].

There is empirical evidence for the evolution of reproductive isolation under sympatric conditions [3,15-19], however, sympatric speciation remains difficult to prove because reproductive isolation could as well have originated during an allopatric period, followed by secondary contact and introgression ([20], but see [19] and citations herein). To investigate mechanisms of premating isolation under sympatric conditions, Servedio [21] modeled increased linkage between genes controlling traits associated with mate recognition and genes controlling hybrid incompatibilities. She demonstrated that hybrid incompatibilities may develop under circumstances of increased linkage despite some gene flow and state that "postzygotic isolation may build up during the sympatric speciation process, accelerating and easing the conditions for speciation to proceed".

Rice and Hostert [22] showed that hybrids of two ecotypes can suffer reduced fitness, both because they may fall between distinct ecological niches of their parental populations and be selected against (extrinsic postzygotic isolation), and/or because of genetic incompatibilities between the parental populations (intrinsic postzygotic isolation). Negative epistatic effects have been shown to reduce hybrid fitness if populations that have diverged in allopatry meet again secondarily [23-25]. This could be explained by the Dobzhansky-Muller model [26,27]. In the Dobzhansky-Muller model, hybrid sterility and inviability develop as pleiotropic byproducts during independent allopatric divergence of populations. Thereby substitutions, which have accumulated during allopatry and are neutral or beneficial in the genetic background of one population or species, are deleterious in hybrids when the species meet secondarily.

Schluter [28], Hatfield and Schluter [29] and Rundle [30] tested whether extrinsic and/or intrinsic postzygotic isolation played a role in stickleback speciation. They showed that F_1 _hybrids and backcrosses of sympatric populations of sticklebacks, a benthic and a limnetic form, had lower growth rates in both parental habitats. These results were regarded as an indication that extrinsic postzygotic isolation can occur. Intrinsic postzygotic isolation was negligible for both populations of sticklebacks.

**Figure 1:**
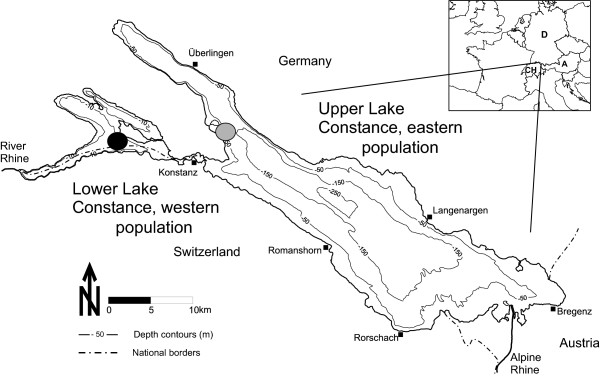
**Map of sampling area**. Lake Constance with the sampling sites for perch (*Perca fluviatilis *L.) belonging to two genetically divergent populations. Black dot, sampling locality for western population; grey dot, sampling locality for eastern population.

Eurasian perch (*Perca fluviatilis *L.) provide a great opportunity to study postzygotic mechanisms that might promote reproductive isolation and drive population divergence between populations. Very recently, Bergek and Björklund [31] have shown that perch populations within a small lake are genetically differentiated between areas that may be less than 1000 m apart. The first evidence for similar population subdivision in perch of Lake Constance was found by analyzing allozyme variation [32], and differences in parasite infection rates at different localities around the lake [33]. Motivated by these findings, Gerlach *et al*. [34] investigated the genetic structure of perch using microsatellites and found that they are subdivided into two genetically distinct populations (Genetic index of between population divergence F_ST _= 0.07, [34]). The populations are geographically stratified within the lake. One population inhabits the eastern the other the western part (Fig. 1) while no obvious geographical barrier separates the two [34]. No further genetic sub-structuring or any indication of inbreeding or homing of adults to spawning sites could be detected within the two populations [34]. The last finding was in contrast to tagging experiments by Jarv [35] that indicated a homing behavior for Eurasian perch in the coastal waters of western Estonia.

Lake Constance is a very young prealpine lake, covering an area of 536 km^2 ^that has formed at the end of the last Pleistocene glaciations [36]. The earliest colonization by perch could thus have occurred 15 000 – 10 000 ybp [37]. Perch from the two putative glacial refuges of Lake Constance, the Danube and Rhine rivers, differ in their mtDNA D-loop sequences [38]. To investigate if genetic differentiation of perch from Lake Constance was due to multiple colonization from these two Pleistocene refuges, Behrmann-Godel *et al*. [37] sequenced 365 bp of the 5'end of the mtDNA D-loop. Almost exclusively Danube haplotypes were found within both populations, Rhine haplotypes also occurred in both populations but in very low frequencies. However, no significant population sub-structuring could be observed.

Here we analyze if postzygotic reproductive isolation occurs between the two perch populations of Lake Constance. We tested F_1 _hybrid crosses of both populations for the combined fitness parameters, fertilization rate and hatching success, to investigate whether intrinsic postzygotic isolation could contribute to divergence between these perch populations.

## Results

**Figure 2:**
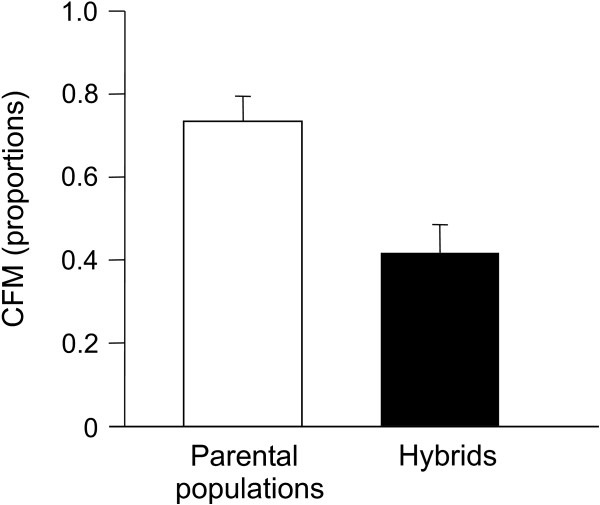
**Reduced fitness of hybrids**. CFM (combined fitness measure, error bars = SE, for details see text) for parental populations and F_1 _hybrid crosses of perch from two sympatric populations in Lake Constance.

The combined fitness measure (CFM) differed significantly between crosses (Table 1, ANOVA on arcsine square root of proportions, F_3,23 _= 5.30, P = 0.006). It was higher in the parental populations (eastern pop, 63%; western pop, 82%) and lower in the hybrid crosses (F_1_1, 51%, F_1_2, 34%). There was no significant difference in the CFM, neither between the two parental crosses (linear contrast analysis, F_1,23 _= 1.79, P = 0.193) nor between the two hybrid crosses (linear contrast analysis, F_1,23 _= 2.27, P = 0.146). We therefore pooled the data for the two hybrid and the two parental crosses. Compared to the parental crosses, hybrid crosses had a significantly lower CFM (Fig. 2, linear contrast analysis, F_1,23 _= 10.28, P = 0.004).

**Table 1:** Various fitness parameters for parental and hybrid crosses

**Crosses**	**Number of females**	**Number of eggs**	**Fertilized**	**Hatched**	**CFM**
**East. pop.**	5	788 (139)	0.953 (0.002)	0.651 (0.108)	0.625 (0.104)
**West. pop.**	6	773 (56)	0.985 (0.009)	0.835 (0.049)	0.824 (0.054)
**F**_1_**1**	7	1030 (61)	0.986 (0.005)	0.517 (0.087)	0.510 (0.086)
**F**_1_**2**	9	917 (71)	0.560 (0.149)	0.507 (0.116)	0.344 (0.098)

Mean (± SE) proportions of fertilized eggs, hatched larvae (from fertilized eggs) and CFM (combined fitness measure, for details see text) of the different crosses. East. pop./West. pop. = crosses within populations. Hybrid crosses F_1_1 = female eastern population/male western population, F_1_2 = female western population/male eastern population.

Fertilization success of eggs differed within our four experimental groups (Table 1, ANOVA on arcsine square root of proportions, F_3,23 _= 4.86, P = 0.009). F_1_2 hybrids had a significantly lower egg fertilization rate as compared to all other crosses (eastern pop, 95%; western pop, 99%, F_1_1, 99%, F_1_2, 56%; linear contrast analysis, F_1,23 _= 14.29, P = 0.001). The hatching success did not differ significantly between crosses (Table 1, ANOVA on arcsine square root of proportions, F_3,23 _= 2.14, P = 0.122).

Six out of nine F_1_2 hybrid crosses were conducted under laboratory conditions. This method may have resulted in reduced hybrid fitness of these particular crosses. However, a statistical analysis excluding these crosses did not alter our results. The CFM between crosses were still significantly different (ANOVA on arcsine square root of proportions, F_3,17 _= 3.60, P = 0.035) and hybrids still had a significantly lower CFM than parental crosses (linear contrast analysis, F_1,17 _= 7.13, P = 0.016 whereas there was no significant difference in the CFM, neither between the two parental crosses (linear contrast analysis, F_1,17 _= 2.82, P = 0.11) nor between the two hybrid crosses (linear contrast analysis, F_1,17 _= 0.12, P = 0.74).

## Discussion

Our results show reduced hybrid fitness in crosses of two genetically divergent populations of perch in Lake Constance which only differ by a mean F_ST _value of 0.07

Both types of hybrid crosses had significantly lower CFM values than the within population crosses indicating intrinsic postzygotic isolation. This could be based on an acceleration of Dobzhansky- Muller's rate of incompatibilities [26,27] by divergent selection which is generally believed to be the basis of most intrinsic genetic incompatibilities [39-41].

**Figure 3:**
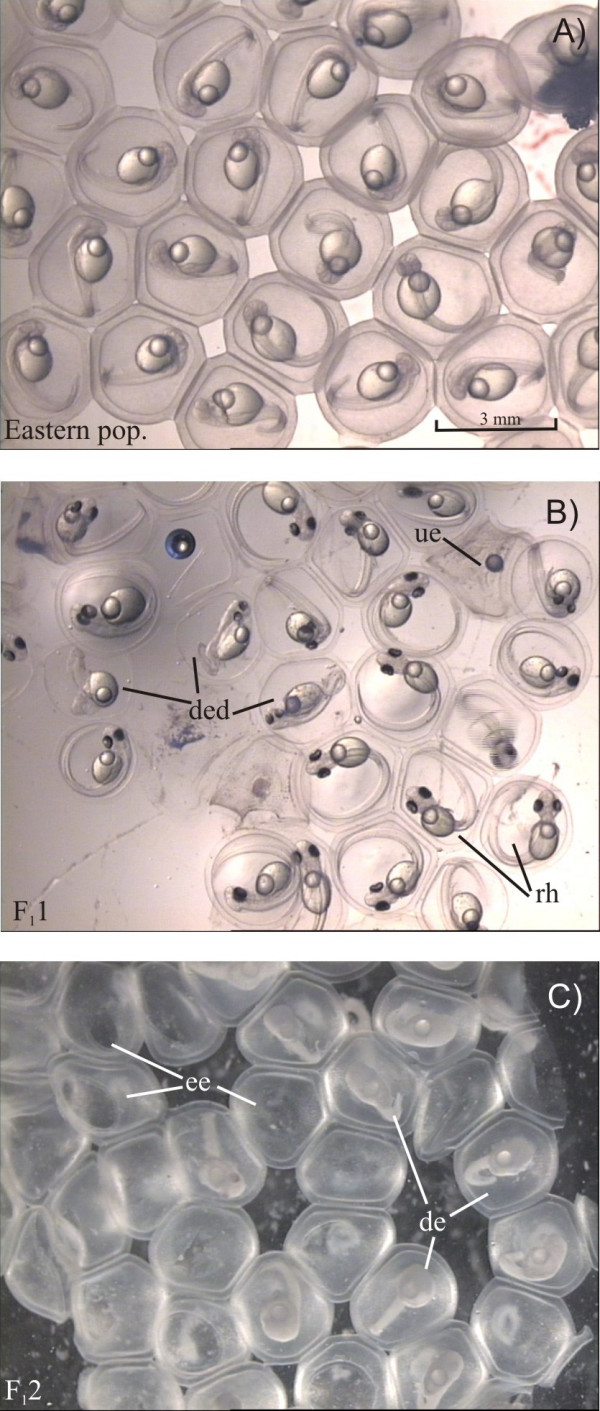
**Embryonic development of hybrids and parental crosses**. Embryonic development of perch from different crosses. A) Embryos from a parental population (eastern population), four days after fertilization. B) Egg-strand of cross F_1_1, female eastern/male western population, 10 days after fertilization. rh = ready to hatch embryos, ded = embryos with disturbed embryonic development, ue = unfertilized eggs. C) Egg-strand of cross F_1_2, female western/male eastern population, one day after hatching. de = dead embryos of different embryonic stages, ee = empty egg shells that remained after hatching.

The comparison of fertilization and hatching success between parental and hybrid crosses (Table 1) indicated an asymmetric crossing barrier, which can occur, if the viability of hybrids differ depending on which species is the maternal parent [42]. Compared to parental populations, perch F_1_2 hybrid crosses (female western population/male eastern population) showed significantly reduced fertilization success; while F_1_1 hybrid crosses (female eastern population/male western population) did not. Such an unidirectional cross-fertilization has already been observed in numerous species, e.g. in fish [42,43], in sea urchins [44-46] in *Drosophila *[47] and in many plant taxa [48]. Additionally, we found a trend towards reduced hatching success of hybrids, indicating additional postzygotic postmating isolation [43]. The decreased hatching success was based on the high mortality rate during embryonic development of hybrid crosses (Fig. 3). Many deformed embryos that died before hatching, were observed in the F_1_1 hybrid cross and at a much lower frequency also in other crosses. Our results indicate that hybridization between eastern and western perch populations in Lake Constance could indeed result in a certain amount of incompatible genotypes in hybrid offspring, contributing to the disruptive divergence of populations [49].

The slight differences (approx. one to two weeks) in the peak spawning times of the two populations could have resulted in crossing early and late breeders in the hybrid but not in the parental crosses. Early and late breeders within populations may be adapted to specific seasonal conditions and their hybrids may therefore exhibit outbreeding depression [50]. However, we do not think that this explains the differences found in CFM values. In our hybrid crosses, all females were caught during peak spawning of their respective population (see methods section). The reproductive period of male perch exceed that of females. Males of both populations held in captivity stay ripe and running and can inseminate eggs from multiple females over a time period of two weeks before and after the main peak spawning time (Behrmann-Godel, personal observation). Therefore, reduced CFM values of hybrid crosses could not have been caused by not yet ripe or already depleted males.

Since we have no further data to clarify whether the observed reduced hybrid fitness evolved in sympatry, parapatry or allopatry, we suggest that the two populations of perch may have diverged during or at the end of the last ice age, which can be dated to 115 000 to 10 000 ybp [37]. This seems a surprisingly short time span for genetic incompatibilities to evolve. Russell [51] and Bolnick and Near [42] have shown that hybrid viability declined with the time separating pairs of different fish species. Based on a Centrarchid phylogeny, calculations of Bolnick and Near [42] revealed that it takes approx. 5–10 my before the hatching compatibility of interspecific crosses starts to decrease. Similar results have been found for species pairs of darters (Percidae: *Etheostoma*) [43] and also for bird species [52], but Price and Bouvier [52]could also show that hybrid infertility arises much earlier (in the order of a few million years). However, at least for allopatric *Drosophila *sister species, mathematical modeling shows that hybrid sterility and inviability evolve fast enough (in the order of thousands of years, assuming single incompatibility speciation) to contribute to speciation [39].

Our results on reduced hybrid fitness in perch are supported by findings of Lu and Bernatchez [53] and Rogers and Bernatchez [54] who showed that intrinsic and extrinsic postzygotic reproductive isolation in lake whitefish (*Coregonus culpeaformis*) could promote speciation. In hybrid-parental backcrosses between two allopatric populations of whitefish, Rogers and Bernatchez [54] found increased embryonic mortality and asynchrony in the developmental time to emergence of backcrosses. This indicated both, intrinsic and extrinsic mechanisms contributing to the formation and maintenance of reproductive isolation between lake whitefish populations that have diverged during the last Pleistocene glaciations, 18 000 to 50 0000 ybp, which is a quite comparable time span to the divergence time we suggested for the two perch populations of Lake Constance.

Coyne and Orr [55] showed that in *Drosophila *sister species prezygotic sexual isolation is stronger than postzygotic isolation, and that in sympatric species prezygotic isolation is greatly enhanced in comparison to allopatric species. We might expect to find prezygotic isolation mechanisms prevalent in the perch populations of Lake Constance as well. The geographical subdivision of the two populations resulting in slight differences of their peak spawning times could act to produce the early state of prezygotic isolation. This could be enhanced by mate choice decisions, leading to assortative mating [56] and further be promoted by postzygotic isolation based on reduced hybrid fitness as shown in this study. We showed earlier [56] that based on olfactory cues juvenile perch prefer their own over the other population. This indicates that a mechanism exists to differentiate between populations that could also be used to avoid mating partners of the other population. Therefore, prezygotic isolation mechanism such as population assortative mating may exist, which could be under positive selection due to reduced hybrid viability.

Since we used artificial fertilization in our laboratory experiments, we do not know whether perch will hybridize under natural conditions. So far, we have detected only slight morphological differences between the two populations (Behrmann-Godel, unpublished). Thus, it is not possible to identify parental populations and hybrids based on morphological parameters in order to survey the lake for the existence of hybrids. Genetic assignment tests will show, whether hybrids of both populations exist.

Besides indication for intrinsic postzygotic isolation, we suggest also extrinsic postzygotic isolation corroborating reproductive isolation of perch populations in Lake Constance. Each population inhabits a lake basin which is geographically connected to the other but differs in trophic state, temperature regime, mean water depth, vegetation, proportion of the littoral zone and many more biotic and abiotic parameters [57]. Each population may be well adapted to the ecological conditions in their part of the lake; however, hybrids may experience lower fitness in either of the two parental habitats.

## Conclusion

In conclusion we found reduced fitness in hybrids between two perch populations inhabiting Lake Constance that may have diverged rather recently, between 115 000 and 10 000 ybp. Our results provide empirical evidence for the evolution of genetic incompatibilities between the two populations that might promote reproductive isolation and drive further population divergence.

## Methods

### Fish sampling and artificial fertilization

Artificial fertilization was used to breed hybrids of the two perch populations of Lake Constance. In the shallower Lower Lake (western population), water temperature increases earlier in spring than in the deeper Upper Lake (eastern population) causing the western population to spawn approximately one to two weeks earlier than the eastern population. For both populations, the entire duration of spawning lasts for about one month, but most fish reproduce within one week [58]. During peak spawning of the western population, gill nets were exposed overnight at two to three different localities (approximately 100 to 500 m apart) in both the eastern and western part of the lake (Fig. 1). Early the next morning males of the eastern population were carefully removed from the gill nets and transported alive to the western part of the lake. Females and males of the western population were removed from the gill nets and females were stripped immediately. For the hybridization experiments, egg-strands from western females were fertilized with either the sperm of a male from the western population (parental cross) or from the eastern population (hybrid cross). Six days later, egg-strands from eastern females received the same treatment, but in the reverse direction. Each fish was crossed only once. This procedure resulted in four different crosses, parental crosses from the eastern (N = 5) and western (N = 6) population, a hybrid cross F_1_1 using females from the eastern and males from the western population (N = 7), and a hybrid cross F_1_2 using females from the western and males from the eastern population (N = 9).

We did not initially catch sufficient numbers of ripe females from the western population to conduct all F_1_2 hybrid crosses directly in the field. Therefore, we took seven nearly ripe western females and ripe eastern males to the laboratory. Females were kept in a 250 liter aquarium (10°C, continuous flow of lake water), males were kept in aerated water bottles. Since females may become ripe and running within a few hours, we checked the females twice a day by applying slight pressure on the abdomen. As soon as a female could be stripped (3 females on the day following capture, 3 other females during the following 5 days) the egg-strand was fertilized with the sperm of a male from the eastern population.

### Breeding and acquisition of data

Fertilized egg-strands were incubated in separate tanks (9 liters, constant supply of tap water at 0.1 liter min^-1^, temperature 11°C, raised to 18°C during the following 4 days, 14 h of light). Three to four days after fertilization the number of eggs in each tank was reduced from several thousands to approximately 1000 by cutting out a piece of the middle part of every egg-strand. The number of unfertilized eggs was counted for each egg-strand piece. One to two days after hatching the number of hatched larvae and dead embryos was counted. These data were used to calculate the initial number of eggs per tank and find the proportion of eggs that were successfully fertilized and the proportion of larvae that successfully hatched from fertilized eggs.

To determine hybrid fitness, we used a combined fitness measure (CFM) according to Hatfield and Schluter [29] by multiplying the proportion of fertilized eggs by the proportion of larvae that had hatched from fertilized eggs.

## Competing interests

The author(s) declare that they have no competing interests.

## Authors' contributions

JBG conceived of the study, participated in its design, the data acquisition and drafted the manuscript. GG participated in the design of the study and the data acquisition. All authors read and approved the final manuscript.
